# Supplementary material for: Providing psychological support to people impacted by terminal illness: A mixed methods study of hospice staff perceived competence, confidence and training needs
Source: PLoS One. 2025 Sep 24;20(9):e0331680. doi: 10.1371/journal.pone.0331680 (PMC12459835; doi:10.1371/journal.pone.0331680)
Supplement: S1 File — (DOCX) [file pone.0331680.s001.docx]

**Understanding current practices regarding how psychological support is provided for terminally ill patients and their caregivers**

**Online Questionnaire**

|  |  | |
| --- | --- | --- |
|  | |  |
|  | |  |
|  | |  |
|  | |  |
|  | |  |
|  | |  |
|  | |  |
|  | |  |

***Section 1: Assessing and recognising psychological support needs***

1. **In your experience what proportion of patients would present with psychological or psychosocial distress**

76-100% (i.e. over three-quarters of all patients)

51-75% (i.e. between half and three-quarters of all patients)

26-50% (i.e. one quarter to a half of all patients)

0-25% (i.e. Up to one-quarter of all patients)

1. **Which of the following most closely applies to how you often you assess your patients’ psychological support needs**

- On first assessment
- Regularly
- Occasionally
- Rarely
- Other (please describe) ___________

1. **What are the common psychological concerns of the patients/carers you are trying to help? (select all that apply)**

- Anxiety
- Depression or low mood
- Adjustment to their diagnosis
- Relationship difficulties
- Other____________________________________________

1. **In general, how easy or difficult is it to assess if a patient is experiencing psychological or psychosocial distress**

- Easy
- Fairly easy
- Neither easy nor difficult
- Fairly difficult
- Difficult

1. **What factors influence how easy or difficult it is to assess psychological or psychosocial distress. Please list any you can think of below. [open question]**
2. **Please indicate how much you agree with the following statements:**

|  | **Strongly Agree** | **Somewhat Agree** | **Somewhat Disagree** | **Strongly Disagree** |
| --- | --- | --- | --- | --- |
| For new patients, the external referrer usually provides information about any psychological or psychosocial concerns (eg via letter, in patient record, or via call) |  |  |  |  |
| I am comfortable initiating conversations around psychological or psychosocial distress with patients and/or their caregivers |  |  |  |  |
| I am confident in my ability to recognise psychological distress in patients and carers) |  |  |  |  |
| I am confident in my ability to differentiate mild, moderate and severe psychological needs in my patients |  |  |  |  |
| I believe formal screening tools (e.g., a distress thermometer etc.) are helpful when screening for patient or caregiver distress |  |  |  |  |
| I feel confident using formal screening tools (e.g., a distress thermometer etc.) to screen for patient and/or caregiver distress |  |  |  |  |
| I can recognise signs of appropriate psychological distress as well as psychological / psychosocial distress that may need more urgent attention / intervention |  |  |  |  |
| I am competent in differentiating between distress as a normal response to a diagnosis of terminal illness and distress that requires referral to a counsellor, clinical psychologist or mental health specialist |  |  |  |  |
| I have received sufficient training in psychological needs assessment |  |  |  |  |

1. **Do you ever use formal assessment tools to assess psychological or psychosocial need of patients and/or their caregivers?**

- Never
- Sometimes
- Often
- Always

1. **If you use formal tools to assess psychological or psychosocial needs, please indicate which one(s) below [Open Question]**

1. **How do you informally assess how a person experiences their situation with regard to their psychological wellbeing. (Open Question)**

***Section 2 Management of psychological support needs***

1. **Please indicate how much you agree with the following statements:**

|  | **Strongly Agree** | **Somewhat Agree** | **Somewhat Disagree** | **Strongly Disagree** |
| --- | --- | --- | --- | --- |
| I routinely provide patients and their caregivers with information to help them understand their psychological symptoms. |  |  |  |  |
| I am able to openly discuss distressing issues with the patient and/or their caregivers and discuss ways in which we might manage and hopefully alleviate their distress. |  |  |  |  |
| I regularly provide space and opportunity to allow a patient and/or their caregivers to express how they feel. |  |  |  |  |
| I feel confident in my ability to pick up on subtle ques, give gentle prompts and provide space for exploration of anxieties. |  |  |  |  |
| I am not afraid to explore difficult subject matter (Death and dying) |  |  |  |  |
| I feel able to discuss sensitive subject matter regarding a patient’s psychological and/or psychosocial concerns |  |  |  |  |
| I can provide patients and their caregivers with information about options for psychological support available to them |  |  |  |  |
| I am confident in my ability to support patients or families to manage their distress |  |  |  |  |
| I am able to facilitate communications about psychological concerns between the patient and family in order to achieve the best outcome for patient and family. |  |  |  |  |

1. **What kinds of patient or family related complications might make it difficult for you to provide psychological support to a terminally ill person e.g., fatigue, cognitive issues such as poor concentration, understanding, memory, reluctance to engage, denial (Open Question)**

1. **What kinds of organisation-based issues make it difficult for you to provide psychological support, e.g., the daily routine, staffing issues, other responsibilities (Open Question)**

1. **Which psychological support needs do you find most difficult to manage, and why?**   **(Open Question)**

1. **What are some differences between providing psychological or psychosocial support to patients versus their families or caregivers?**   **(Open Question)**

***Section 3 Referral for specialist psychological support***

1. **Please indicate how much you agree with the following statements:**

|  | **Strongly Agree** | **Somewhat Agree** | **Somewhat Disagree** | **Strongly Disagree** |
| --- | --- | --- | --- | --- |
| I can arrange appropriate psychological support for my patients when I need to |  |  |  |  |
| There are clear guidelines regarding referral for specialist psychological support within my organisation |  |  |  |  |

1. **If a patient requires more psychological support than I can provide, I can refer the patient Internally (i.e.  In the location where I work) to the following (tick all that apply):**

- Clinical Psychologist (based in the location where I work)
- Social worker
- Doctor or nurse trained in providing psychological support
- Allied health professional trained in providing psychological support
- Psychological support provided by day therapies
- Counsellor
- Bereavement Counsellor
- Complementary therapist
- Other _______________________________

1. **If a patient requires more psychological support than I can provide, I can refer the patient externally to the following (tick all that apply):**

- Clinical Psychologist
- Social worker
- Doctor or nurse trained in providing psychological support
- Allied health professional trained in providing psychological support
- Psychological support provided by day therapies
- Counsellor
- Bereavement Counsellor
  - Complementary therapist
- Psychiatrist
- Other _________________________________

1. **Is there anything that you feel could be done to improve referral procedures within your workplace?** (Open Question)

***Section 4 Education and training***

1. ***Have you received any formal or informal training in providing psychological support to terminally ill patients and their families?***

- Yes
- No

***19b [If yes to previous question] If* you have received training focused on providing psychological support has this training been**

- Informal on the job training (e.g., through discussion with colleagues)
- In-house training as part of a general training day on a range of topics
- In-house training focused on psychological support
- External training day or workshop focused on psychological support
- A short, unaccredited course over a week or similar
- Comprehensive, longer-term training, such as a certificate or diploma in psychological support/interventions
- Other_____________________________________________

**Please add detail if appropriate**

**19c Please indicate if you have received any certified training in the following (tick all that apply):**

- Cognitive behavioural therapy (CBT)
- Acceptance and commitment therapy (ACT)
- Narrative therapy
- Compassion-Focused therapy
- Mindfulness strategies
- Psychodynamic approaches
- Systemic therapy
- Hypnotherapy
- Music therapy
- Art therapy
- Dignity therapy
- Managing Cancer and Living Meaningfully (CALM)
- Meaning-Centred Psychotherapy
- Supportive-Expressive Group therapy (SEGT)
- Problem-Solving therapy
- Solution-Focused therapy
- Counselling
- **Other** ______________________

1. **Please indicate how much you agree with the following statements:**

|  | **Strongly Agree** | **Somewhat Agree** | **Somewhat Disagree** | **Strongly Disagree** |
| --- | --- | --- | --- | --- |
| I have received sufficient training in how to meet the psychological support needs of **patients** with a terminal illness |  |  |  |  |
| I have received sufficient training in how to meet the psychological support needs of **caregiver**s of patients with a terminal illness |  |  |  |  |
| I feel sufficiently trained to deliver effective psychological support to my patients and their caregivers |  |  |  |  |
| Training in psychological support would enhance my practice |  |  |  |  |
| I receive useful advice, support or feedback from my manager/supervisor regarding psychological support for patients and caregivers, as required. |  |  |  |  |
| I receive useful advice, support, or feedback from other experienced staff regarding providing psychological support for patients and caregivers, as required. |  |  |  |  |

1. **Is there anything that you feel would further improve your confidence in delivering psychological support to your patients and/or their caregivers?**

***Final background Questions***

1. **Which of the following best describes your role?**

- Nurse
- Community Palliative Care Clinical Nurse Specialist
- Doctor
- Consultant
- Healthcare assistant
- Pharmacist
- Occupational Therapist
- Physiotherapist
- Social worker
- Chaplain
- Bereavement counsellor
- Other

If other, please state: ____________________________________

1. **Do you manage or supervise one or more members of staff?**
   - Yes
   - No
2. **How long have you worked with terminally ill patients?**

- <1 year
- 1-5 years
- 6-10 years
- >11 years

1. **Which setting best describes your work setting**

Inpatient unit (Hospice or hospital palliative care inpatient unit)

Community setting (provide care to people at home and in care homes)

Outpatient/day therapies

Care home only

Other - Pls describe: ___________________

1. **Are you:**

- Female
- Male
- Non-binary
- Prefer not to say
- Prefer to add my own description______________________________

1. **What age are you?**   ________ (years)
2. **What is your race/ethnicity?**

_________________________________

1. **In which UK region/country do you work:**
   - Scotland
   - Northern Ireland
   - England
   - Wales
   - Republic of Ireland

**You have now completed this questionnaire.**

**Your participation has been vital in helping us understand how psychological support is offered to terminally ill patients and their caregivers, and may help inform future practice in this area.**

**If you would like to hear about the results, please email** [**XXXX@ed.ac.uk**](mailto:XXXX@ed.ac.uk) **and we will email you with a summary once we have completed the study.**

**Thank you so much for your time.**

**I**
