# Supplementary material for: Providing psychological support to people impacted by terminal illness: A mixed methods study of hospice staff perceived competence, confidence and training needs
Source: PLoS One. 2025 Sep 24;20(9):e0331680. doi: 10.1371/journal.pone.0331680 (PMC12459835; doi:10.1371/journal.pone.0331680)
Supplement: S2 File — (DOCX) [file pone.0331680.s002.docx]

**Supplementary File 2**

**Self-reported competence and confidence in providing psychological support**

Scoring- A score of self-perceived competence and confidence in providing psychological support was developed based on participants’ responses to the following items:

**Competence in providing psychological support**

1. I can recognise signs of appropriate psychological distress as well as psychological / psychosocial distress that may need more urgent attention / intervention
2. I am competent in differentiating between distress as a normal response to a diagnosis of terminal illness and distress that requires referral to a counsellor, clinical psychologist or mental health specialist
3. I am able to openly discuss distressing issues with the patient and/or their caregivers and discuss ways in which we might manage and hopefully alleviate their distress
4. I feel able to discuss sensitive subject matter regarding a patient’s psychological and/or psychosocial concerns
5. I can provide patients and their caregivers with information about options for psychological support available to them
6. I am able to facilitate communications about psychological concerns between the patient and family in order to achieve the best outcome for patient and family.
7. I can arrange appropriate psychological support for my patients when I need to
8. I feel sufficiently trained to deliver effective psychological support to my patients and their caregivers

**Confidence** **in providing psychological support**

1. I am comfortable initiating conversations around psychological or psychosocial distress with patients and/or their caregivers
2. I am confident in my ability to recognise psychological distress in patients and carers)
3. I am confident in my ability to differentiate mild, moderate and severe psychological needs in my patients
4. I feel confident using formal screening tools (e.g., a distress thermometer etc.) to screen for patient and/or caregiver distress
5. I feel confident in my ability to pick up on subtle ques, give gentle prompts and provide space for exploration of anxieties.
6. I am not afraid to explore difficult subject matter (Death and dying)
7. I am confident in my ability to support patients or families to manage their distress

**Competence scores by item**

|  | **Strongly Agree**  **(%, n)** | **Somewhat Agree**  **(%, n)** | **Somewhat Disagree**  **(%, n)** | **Strongly Disagree**  **(%, n)** |
| --- | --- | --- | --- | --- |
| I can recognise signs of appropriate psychological distress as well as psychological / psychosocial distress that may need more urgent attention / intervention | 36%  55 | 56%  85 | 6%  9 | 1%  2 |
| I am competent in differentiating between distress as a normal response to a diagnosis of terminal illness and distress that requires referral to a counsellor, clinical psychologist or mental health specialist | 32%  49 | 52%  79 | 12%  18 | 3%  5 |
| I am able to openly discuss distressing issues with the patient and/or their caregivers and discuss ways in which we might manage and hopefully alleviate their distress. | 47%  71 | 48%  73 | 5%  7 | 0%  0 |
| I feel able to discuss sensitive subject matter regarding a patient’s psychological and/or psychosocial concerns | 55%  83 | 40%  61 | 4%  6 | 1%  1 |
| I can provide patients and their caregivers with information about options for psychological support available to them   (N=150) | 38%  57 | 44%  66 | 15%  23 | 3%  4 |
| I am able to facilitate communications about psychological concerns between the patient and family in order to achieve the best outcome for patient and family (N=150) | 36%  54 | 50%  75 | 13%  19 | 1%  2 |
| I can arrange appropriate psychological support for my patients when I need to | 30%  45 | 34%  51 | 27%  41 | 9%  14 |
| I feel sufficiently trained to deliver effective psychological support to my patients and their caregivers | 16%  24 | 36%  53 | 38%  57 | 10%  15 |

**Confidence scores by item**

|  | **Strongly Agree**  **(%, n)** | **Somewhat Agree**  **(%, n)** | **Somewhat Disagree**  **(%, n)** | **Strongly Disagree**  **(%, n)** |
| --- | --- | --- | --- | --- |
| I am comfortable initiating conversations around psychological or psychosocial distress with patients and/or their caregivers | 58%  87 | 40%  60 | 2%  3 | 1%  1 |
| I am confident in my ability to recognise psychological distress in patients and carers (N=150) | 45%  68 | 53%  79 | 2%  3 | 0%  0 |
| I am confident in my ability to differentiate mild, moderate and severe psychological needs in my patients | 25%  38 | 60%  91 | 13%  19 | 1%  2 |
| I feel confident using formal screening tools (e.g., a distress thermometer etc.) to screen for patient and/or caregiver distress | 13%  19 | 32%  47 | 42%  63 | 13%  20 |
| I feel confident in my ability to pick up on subtle ques, give gentle prompts and provide space for exploration of anxieties. | 53%,  80 | 44%  66 | 3%  5 | 0%  0 |
| I am not afraid to explore difficult subject matter (Death and dying) | 72%  108 | 25%  38 | 3%  5 | 0%  0 |
| I am confident in my ability to support patients or families to manage their distress | 32%  48 | 57%  86 | 11%  17 | 0%  0 |
