# Supplementary material for: Providing psychological support to people impacted by terminal illness: A mixed methods study of hospice staff perceived competence, confidence and training needs
Source: PLoS One. 2025 Sep 24;20(9):e0331680. doi: 10.1371/journal.pone.0331680 (PMC12459835; doi:10.1371/journal.pone.0331680)
Supplement: Attachment — Submitted filename: PONE-D-25-05121_reviewer edits_10APR2025.pdf [file pone.0331680.s004.pdf]

PLOS ONE

Providing psychological support to people impacted by terminal illness: A questionnaire study of hospice staff confidence and training needs

--Manuscript Draft--

**Manuscript Number:**	PONE-D-25-05121
**Article Type:**	Research Article
**Full Title:**	Providing psychological support to people impacted by terminal illness: A questionnaire study of hospice staff confidence and training needs
**Short Title:**	Psychological support in hospices
**Corresponding Author:**	Anne Finucane, PhD<br>The University of Edinburgh<br>Edinburgh, Scotland UNITED KINGDOM OF GREAT BRITAIN AND NORTHERN IRELAND
**Keywords:**	Hospice Care, staff development, professional competence, psychosocial support systems, psychological support systems
**Abstract:**	Background<br>Psychological distress is common amongst people with a terminal illness. While mental health specialists have a role in assessment and management of those with complex psychological problems, palliative care clinicians provide psychological support to most patients and families.<br>Aim<br>To describe current practices relating to the provision of psychological support by hospice clinicians, and to explore confidence and training needs in relation to this aspect of their role.<br>Design<br>An online questionnaire consisting of closed and free-text questions, emailed to 273 hospices in the UK and Republic of Ireland between May and June 2023.<br>Setting/Participants<br>Eligible respondents included nurses, doctors and allied health professionals employed by a hospice. Quantitative data was analysed descriptively using SPSS 27, open-text data was analysed thematically guided by a framework approach.<br>Results<br>151 hospice staff completed the questionnaire. Nearly all respondents assess patients for psychological distress, but clinical judgement was most common and 58% never used a validated assessment tool. Respondents reported high levels of confidence and competence in providing psychological support especially in recognizing psychological distress and openly discussing distressing topics. Confidence and competence was lower in relation to screening for psychological support needs and referring onwards to mental health specialists. Overall, 72% reported that training in psychological support would further enhance their practice. Individual and family factors such as denial, communication challenges and family conflict made it more difficult to provide psychological support. Systemic factors were time constraints, prioritisation of physical symptoms and limited access to mental health specialists.<br>Conclusion<br>Palliative care staff report that they are confident in providing basic psychological support, however there was a desire for further training in this aspect of care. Clearer guidance on referral criteria for specialist psychological support is warranted.
**Order of Authors:**	Fatema Zavery
	Sulafa Fakhreldin
	Sarah Huibregtse Van Loon
	Mhairi McMcDougall
	Anne Finucane, PhD
**Additional Information:**	

Powered by Editorial Manager® and ProduXion Manager® from Aries Systems Corporation

<table>
<tr><th>Question</th><th>Response</th></tr>
<tr><td>Financial Disclosure

Enter a financial disclosure statement that describes the sources of funding for the work included in this submission. Review the submission guidelines for detailed requirements. View published research articles from PLOS ONE for specific examples.

This statement is required for submission and will appear in the published article if the submission is accepted. Please make sure it is accurate.

Funded studies
Enter a statement with the following details:
• Initials of the authors who received each award
• Grant numbers awarded to each author
• The full name of each funder
• URL of each funder website
• Did the sponsors or funders play any role in the study design, data collection and analysis, decision to publish, or preparation of the manuscript?

Did you receive funding for this work?</td><td>The author(s) received no specific funding for this work.</td></tr>
<tr><td>Competing Interests

Use the instructions below to enter a competing interest statement for this submission. On behalf of all authors, disclose any competing interests that could be perceived to bias this work—acknowledging all financial support and any other relevant financial or non-financial competing interests.

This statement is required for submission and will appear in the published article if the submission is accepted. Please make sure it is accurate and that any funding sources listed in your Funding Information later in the submission form are also</td><td>The authors have declared that no competing interests exist.</td></tr>
</table>

Powered by Editorial Manager® and ProduXion Manager® from Aries Systems Corporation

declared in your Financial Disclosure statement.<br><br>View published research articles from *PLOS ONE* for specific examples.<br><br>**NO authors have competing interests**<br><br>Enter: *The authors have declared that no competing interests exist.*<br><br>**Authors with competing interests**<br><br>Enter competing interest details beginning with this statement:<br><br>*I have read the journal's policy and the authors of this manuscript have the following competing interests: [insert competing interests here]*<br><br>* typeset	
**Ethics Statement**<br><br>Enter an ethics statement for this submission. This statement is required if the study involved:<br><br>• Human participants<br>• Human specimens or tissue<br>• Vertebrate animals or cephalopods<br>• Vertebrate embryos or tissues<br>• Field research<br><br>Write "N/A" if the submission does not require an ethics statement.<br><br>General guidance is provided below. Consult the submission guidelines for detailed instructions. **Make sure that all information entered here is included in the Methods section of the manuscript.**	The University of Edinburgh's Health in Social Science ethics committee granted ethical approval on May 11th, 2023. The ethics Case ID for the project is 22-23CLPS072. . Consent for participation was obtained via an online consent form. The online consent form appeared to participants when they clicked on the link to access the online questionnaire. Participants could not proceed to the questionnaire unless they had agreed to each item noted on the consent form.

Powered by Editorial Manager® and ProduXion Manager® from Aries Systems Corporation

**Format for specific study types**<br><br>**Human Subject Research (involving human participants and/or tissue)**<br>• Give the name of the institutional review board or ethics committee that approved the study<br>• Include the approval number and/or a statement indicating approval of this research<br>• Indicate the form of consent obtained (written/oral) or the reason that consent was not obtained (e.g. the data were analyzed anonymously)<br><br>**Animal Research (involving vertebrate animals, embryos or tissues)**<br>• Provide the name of the Institutional Animal Care and Use Committee (IACUC) or other relevant ethics board that reviewed the study protocol, and indicate whether they approved this research or granted a formal waiver of ethical approval<br>• Include an approval number if one was obtained<br>• If the study involved *non-human primates*, add *additional details* about animal welfare and steps taken to ameliorate suffering<br>• If anesthesia, euthanasia, or any kind of animal sacrifice is part of the study, include briefly which substances and/or methods were applied<br><br>**Field Research**<br><br>Include the following details if this study involves the collection of plant, animal, or other materials from a natural setting:<br>• Field permit number<br>• Name of the institution or relevant body that granted permission	
**Data Availability**<br><br>Authors are required to make all data underlying the findings described fully available, without restriction, and from the time of publication. PLOS allows rare exceptions to address legal and ethical concerns. See the PLOS Data Policy and FAQ for detailed information.	Yes - all data are fully available without restriction

A Data Availability Statement describing where the data can be found is required at submission. Your answers to this question constitute the Data Availability Statement and **will be published in the article**, if accepted.<br><br>**Important:** Stating 'data available on request from the author' is not sufficient. If your data are only available upon request, select 'No' for the first question and explain your exceptional situation in the text box.<br><br>Do the authors confirm that all data underlying the findings described in their manuscript are fully available without restriction?	
**Describe where the data may be found in full sentences. If you are copying our sample text, replace any instances of XXX with the appropriate details.**<br><br>• If the data are **held or will be held in a public repository**, include URLs, accession numbers or DOIs. If this information will only be available after acceptance, indicate this by ticking the box below. For example: *All XXX files are available from the XXX database (accession number(s) XXX, XXX.).*<br>• If the data are all contained **within the manuscript and/or Supporting Information files**, enter the following: *All relevant data are within the manuscript and its Supporting Information files.*<br>• If neither of these applies but you are able to provide **details of access elsewhere**, with or without limitations, please do so. For example:<br><br>*Data cannot be shared publicly because of [XXX]. Data are available from the XXX Institutional Data Access / Ethics Committee (contact via XXX) for researchers who meet the criteria for access to confidential data.*<br><br>*The data underlying the results presented in the study are available from (include the name of the third party*	All XXX files are available from the XXX database (accession number(s) XXX, XXX.).

and contact information or URL).<br>• This text is appropriate if the data are owned by a third party and authors do not have permission to share the data.<br><br>* typeset	
Additional data availability information:	Tick here if the URLs/accession numbers/DOIs will be available only after acceptance of the manuscript for publication so that we can ensure their inclusion before publication.

Manuscript

Click here to access/download;Manuscript;PLOS One Submitted 30.01.25.docx

Providing psychological support to people impacted by terminal illness: A questionnaire study of hospice staff confidence and training needs

Fatema Zavery[1¶], Sulafa Fakhreddin[1¶], Sarah Huibregtse Van Loon[1&], Mhairi McDougall[1&], Anne Finucane[1,2*¶]

[1] Clinical Psychology, School of Health in Social Science, University of Edinburgh, Edinburgh, United Kingdom

[2] Marie Curie Hospice Edinburgh, Edinburgh, United Kingdom

*Corresponding author:

Email: a.finucane@ed.ac.uk (AF)

[¶]These authors contributed equally to this work.

[&]These authors also contributed equally to this work.

FZ and SF are joint first authors.

Abstract

Background

Psychological distress is common amongst people with a terminal illness. While mental health specialists have a role in assessment and management of those with complex psychological problems, palliative care clinicians provide psychological support to most patients and families.

Aim

To describe current practices relating to the provision of psychological support by hospice clinicians, and to explore confidence and training needs in relation to this aspect of their role.

Design

An online questionnaire consisting of closed and free-text questions, emailed to 273 hospices in the UK and Republic of Ireland between May and June 2023.

Setting/Participants

Eligible respondents included nurses, doctors and allied health professionals employed by a hospice. Quantitative data was analysed descriptively using SPSS 27, open-text data was analysed thematically guided by a framework approach.

Results

151 hospice staff completed the questionnaire. Nearly all respondents assess patients for psychological distress, but clinical judgement was most common and 58% never used a validated assessment tool. Respondents reported high levels of confidence and competence

in providing psychological support especially in recognizing psychological distress and openly discussing distressing topics. Confidence and competence was lower in relation to screening for psychological support needs and referring onwards to mental health specialists. Overall, 72% reported that training in psychological support would further enhance their practice. Individual and family factors such as denial, communication challenges and family conflict made it more difficult to provide psychological support. Systemic factors were time constraints, prioritisation of physical symptoms and limited access to mental health specialists.

Palliative care does not equal hospice care and should be more clearly discussed in the entire document.

Conclusion

Palliative care staff report that they are confident in providing basic psychological support, however there was a desire for further training in this aspect of care. Clearer guidance on referral criteria for specialist psychological support is warranted.

Need to explain Hospice clinicians provide palliative care to their patients and patients' loved ones. As palliative care encompases more than just Hospice care.

Introduction

Palliative care aims to improve the quality of life of people impacted by life-threatening or terminal illness by attending to their physical, psychological, social and spiritual care needs. Managing pain and physical symptoms is essential, however, psychological and non-physical concerns can equally affect patients' quality of life.[1] Psychological concerns are common amongst people faced with terminal illness and range from general distress to clinically diagnosed psychological problems.[1-4] Psychological distress, including feelings of sadness, anxiety, anger, frustration, fear or worry, is commonly experienced following the diagnosis of a life-limiting illness.[5-7] A retrospective review of referral documentation relating to patients referred to a UK hospice service found that psychological distress was documented for 59% of those being referred.[4] Serious mental health problems are common, and 29%, or one in every three or four patients will experience clinically significant levels of depression, anxiety, adjustment disorder or low mood [2]

International guidelines emphasize the importance of psychological support for people with a terminal illness and offer guidance on its provision.[8-10] One of the most frequently cited models, is that developed in the UK by the National Institute for Health and Care Excellence.[8] This is a four-level model describing recommendations for psychological assessment and support for people with cancer, including advanced cancer. It specifies the appropriate psychological skills, techniques and interventions that could be offered by health and social care professionals depending on the patient's level of need. (Fig 1). In the United States, clinical practice guidelines for quality palliative care from the National Coalition for Hospice and Palliative Care [10] highlight the role of the interdisciplinary team in addressing psychological and psychiatric aspects of care for those with a serious illness. While the social worker plays a key role, all clinicians providing palliative care are expected to have the skills

One

to identify and treat basic psychological conditions such as depression resulting from illness. In Australia, guidelines recommend a 0.15 full-time equivalent staff member from psychology working across community-based services, acute settings and palliative care.[11]

Fig 1. Recommended model of professional psychological assessment and support

Despite the recognition of psychological care as an important domain of palliative care, research evidence on the provision of psychological support in palliative care contexts is lacking. The small number of existing studies reveal broadly similar findings. Access to support for people with significant mental health problems or mental health specialist support needs is insufficient.[12-15]

All health and social care professionals have a role to play in providing basic psychological and emotional support to the patient and their caregivers.[8] Health and social care professionals are typically the first to come in contact with the distress experienced by patients and caregivers and play a key role in identifying and managing anxiety, alongside other members of the multidisciplinary team.[16, 17] In the UK, a survey of hospices in 2019 found that 75% of respondents reported that basic psychological support for patients was being wholly met. However, only 51% believed that needs in relation to screening and problem-solving (NICE Level 2) were being met.[12] Many survey respondents had a management role or led a service providing psychological support, so their views may not be representative of doctors, nurses and allied health professionals in general. Another survey study administered to staff in UK hospices in 2020 found that 25% of respondents did not screen or assess patients for psychological problems, and that two-thirds of nurses and allied

Define Acronym 1st use

health professionals did not feel competent in providing emotional support information at the required level or providing information on the range of treatments available.[14]

To provide high quality palliative care, health and social care staff caring for people with a life-limiting illness need to be confident and competent in their abilities to provide psychological care. Competence refers to the skills, knowledge and abilities to perform successfully or efficiently.[18] Confidence is one's appraisal or feeling of self-assurance arising from their ability to successfully perform a task or achieve an objective and can change as the situation or context changes.[19] It is useful to consider both competence and confidence as this allows a more comprehensive evaluation of an individual's education and training needs.[19]

To inform the future development of psychological support for people impacted by life-limiting illness, up-to-date evidence on the role, confidence and competence of hospice staff in providing psychological support is warranted. Consequently, we sought to investigate the type of psychological support provided by hospice staff who are not employed as mental health specialists; and to explore any further training needs they perceive they may have in relation to this aspect of their role.

Methods

Design

We used a convergent mixed method research design which entails the simultaneous collection of qualitative and quantitative data.[20] We used the questionnaire variant of the convergent mixed methods design which involves using a questionnaire with both closed-

ended and open-ended questions but does not include a complete qualitative data collection.[21] This was a pragmatic decision, enabling the collection of both data types to understand the phenomenon of interest within one study. This design allowed for further elaboration and interpretation of quantitative data based on qualitative findings. This paper is reported in line with the Checklist for Reporting Results of Internet E-Surveys (CHERRIES) guidelines.[22]

Participants

Eligible respondents included health and social care professionals involved in providing direct care to terminally ill patients. We excluded volunteer staff, mental health professionals, and staff affiliated with NHS-based hospices (as we did not have approval to recruit the latter).

Having cancer does not equal Hospice - this needs to be expained more

1st use needs acronym defined

Questionnaire development

We developed an online questionnaire informed by NICE guidelines on improving supportive and palliative care for adults with cancer.[23] The questionnaire draft was screened and refined by a hospice-based clinical psychologist, three community palliative care clinical nurse specialists, a research nurse, and a public representative. Twenty-one questions were chosen for the final questionnaire to fit a timeframe of 10-15 minutes. The final questionnaire comprised four sections: (i) the management of psychological needs (ii) barriers to recognising and assessing psychological support needs, (iii) referral for specialist psychological support and (iv) education and training needs. The final section consisted of questions on demographic information. See Supplementary File 1.

Participant recruitment

Study information was sent to contacts at 273 hospices compiled from the Hospice UK and Irish Association for Palliative Care websites. An invitation email was sent to specific hospice managers asking them to share the study information with clinical staff. The manager's details were found by checking each hospice's website. The email contained the participant information sheet, and a link to the online consent form and online questionnaire. One reminder email was sent after two weeks. Additionally, the research was advertised as a flyer created by the project team on social media which contained a link to the questionnaire. Respondents could enter a prize draw to win one of five £30 vouchers by emailing the study team on completion of the survey. This way, the respondent's name and contact details were separate from their questionnaire responses. Recruitment occurred from 15 May 2023 to 9 June 2023.

Define JISC acronym

Data collection

Questionnaire data was collected through an online questionnaire supported by the JISC Online Surveys platform. The questionnaire was open from May 15th to June 9th, 2023.

Correct name is IBM SPSS Statistics 27

Quantitative data analysis

Quantitative data were analysed using SPSS Version 27. Respondents with significant proportions of missing data (i.e. over 50%) were removed prior to analysis (one respondent). Quantitative data describing current practices was analysed descriptively.

Measures of self-perceived competence and confidence were developed post-hoc during the analysis phase.

Post-hoc self-perceived competence score: A score of self-perceived competence in providing psychological support was developed post-hoc from eight questionnaire items by the research team. This reflected the respondent's self-evaluation of their level of skills in psychological assessment, management, and referral. Scores could range ranged from 8 (self-perception of low competence) to 32 (self-perception of high competence). See Supplementary File 2.

Post-hoc self-confidence score: A score reflecting self-confidence in providing psychological support was developed post-hoc from seven questionnaire items by the research team. This reflected the respondent's beliefs in their capability to perform a task. Scores could range from 7 (low confidence) to 28 (high confidence). See Supplementary File 2.

An independent samples t-test was conducted to investigate differences in mean perceived competence and self-confidence scores between individuals who had and had not received training in providing psychological support. One-way analysis of equal variance (ANOVA) was conducted to examine differences in total perceived competence/confidence scores by role (nurses; doctor/ consultants; allied health professionals or other) and years of experience (>11 years, 6-10 years, 1-5 years, <1 year).

Qualitative data analysis

Data from open-ended questions were analysed using the framework method and examined in relation to pre-existing categories considered relevant to answering the research question: assessment, management, referral and training needs.[24]

Data synthesis

We structured our qualitative and quantitative findings around four pre-determined areas: assessment, management, referral and education and training needs. The presentation of both qualitative and quantitative findings allowed a richer understanding of hospice staff perspectives on how psychological care is provided in practice, with examples of barriers and enablers in relation to providing this care.

Ethical approval

The University of Edinburgh's Health in Social Science ethics committee granted ethical approval on May 11th, 2023. The ethics Case ID for the project is 22-23CLPS072. Consent for participation was obtained via an online consent form. The online consent form appeared to participants when they clicked on the link to access the online questionnaire. Participants could not proceed to the questionnaire unless they had agreed to each item noted on the consent form.

Results

Sample characteristics

152 hospice staff responded. Data from one respondent was excluded as over half of the questions had not been answered, and 151 responses were included in the final analysis. The mean age of respondents was 47 years (SD = 11.4; range 22-72), and 88% were female. Most had over 5 years' experience of working in palliative care, and ove to r half had over 10 years' experience (Table 1). Some missing data was found for 12 respondents, however as most of the questions were completed for these respondents their data was retained for analysis.

over half respondents

Table 1. Sample Characteristics

Characteristics	n	Percentage
Gender identity		
Female	133	88.1%
Male	16	10.6%
Prefer not to say	2	1.3%
Region		
England	98	64.9%
Scotland	21	13.9%
Northern Ireland	16	10.6%
Wales	12	7.9%
Republic of Ireland	4	2.6%
Race/ethnicity		
British	118	82.5%
Indian	1	0.7%
Cornish	1	0.7%

Scottish	9	6.3%
Mixed	2	1.4%
Irish	8	5.6%
European	1	0.7%
Chinese	1	0.7%
Welsh	2	1.4%
Work setting		
Hospice inpatient unit	59	39.1%
Community palliative care	49	32.5%
Across settings	21	13.9%
Outpatient/day therapies	9	6%
Hospital palliative care inpatient unit	5	3.3%
Other	8	5.3%
Role		
Nurse	66	43.7%
Doctor/consultant	31	20.5%

Allied Health Professional (AHP)/chaplain/social work	54	35.8%
Years of Experience		
>11 years	79	52.3%
6-10 years	27	17.9%
1-5 years	35	23.2%
<1 year	9	6%

Not all respondents answered every demographic question. *N*= 146 (Age), *N*= 151 (Gender, Region, Work setting, Role), *N*= 143 (Race/ethnicity), *N*= 150 (Years of Experience).

Perceived prevalence of psychological distress amongst patients receiving palliative care

Most respondents (84%, n=127/151) reported that more than half of all patients they see present with psychological distress. Psychological concerns commonly encountered by staff were anxiety (97%, n= 147/151), adjustment disorder (91%, n=138/151), depression (85%, n=129/151), and relationship difficulties (60%, n=90/151). 'Other' common concerns were noted by 26% of respondents. These included fear of the future, spiritual distress, loss of control, worry about leaving family, and disrupted family dynamics.

Assessment of psychological support needs

Most respondents (81%, n=121/151) reported regularly assessing patients for psychological distress, however only 15% reported that they assess patients' psychological support needs on

first assessment (n = 22/151). Most assessments were based on clinical judgement (58%, n= 87/151), with just under half of respondents using a formal assessment tool. (42%, n= 64/151). Among those using assessment tools, the most used were the Integrated Palliative Care Outcome Scale (IPOS) (42%, n = 26/62) and Hospital Anxiety and Depression Scale (HADS) (32% n=20/62).

Qualitative data revealed barriers to psychological needs assessment: time constraints, denial of diagnosis, communication challenges and the presence of others. Time constraints and clinical pressure meant that staff often need to make quick judgements on psychological support needs:

> *"With significant time constraints and pressures, some colleagues are quick to apply a psychological 'label', rather than sit and talk with the patient" (Respondent 132, Chaplain)*

Denial of a terminal diagnoses, and communication barriers made it difficult for staff to assess psychological distress.

> "*Denial makes assessment difficult.*" (Respondent 41, Clinical nurse specialist)
>
> *"...Level of understanding of patients, language barriers"* [makes it challenging for staff to conduct assessments and provide psychological support] (Respondent 63, Nurse)

The presence of others could also make the assessment of psychological support needs difficult, especially where patients try to mask their distress from those close to them; or where caregivers mask their concerns from the patient.

> *"If family members are present the patient will not always open up"* (Respondent 140, Nurse)

" where a loved one holds back their emotions as not to distress the person who is ill." (Respondent 8, Healthcare assistant)

Management of psychological support needs

The vast majority of respondents indicated that they were competent in the key activities relating to the provision of psychological care (See Supplementary File 2). Self-reported competence was highest in relation to discussing sensitive topics and openly discussing distressing issues and ways to managing this. Self-reported competence was lowest in relation to being able to arrange psychological support and feeling sufficiently trained to deliver effective psychological support. Less than one-third of respondents strongly agreed that they could arrange appropriate psychological support, and only 16% strongly agreeing that they were sufficiently trained to deliver psychological support (Fig 2).

Fig 2. Self-reported staff competence levels in distinct aspects of psychological care (N=151)

The mean *perceived competence* in providing psychological support was 25.2 (SD=5.03). A one-way ANOVA was conducted to identify the effect of role and years of experience on respondents' competence. There was no significant effect of years of experience on respondents' competence score ($F(3,143) = 1.28$, $p= .283$). There was no difference in competence score based on whether the members worked in inpatient or community roles ($F(2,144) = 0.46$, $p= .632$). A t-test was performed to compare perceived competence across individuals who received training and those who did not. Respondents who received any

training (M = 26.56, SD = 3.78), had statistically higher perceived competence scores than those who did not (M = 21.73, SD = 3.57), 95% CI [3.47, 6.18] ($t(145) = 7.04$, $p < 0.01$).

The mean *perceived confidence* in providing psychological support was 22.8 (SD= 3.14). Most respondents 'strongly agreed' or 'somewhat agreed' they were confident in providing key aspects of psychological care (Fig 3). Respondents reported highest levels of confidence in exploring difficult issues and initiating distressing conversations. Lower levels of confidence related to using of formal tools to screen for psychological concerns, with less than half agreeing that they were confident in this. See Supplementary File 2.

Fig 3. Confidence of palliative care staff across various domains of psychological care (N=151)

A one-way ANOVA was conducted to identify the effect of role and years of experience on respondents' confidence. Total confidence scores differed significantly across years of experience ($F(3,144) = 4.43$, $p=.005$). Bonferroni post-hoc test indicated that staff with more than 11 years of experience had higher confidence scores (M=23.45, SD=2.75) than staff with 1-5 years' experience (M= 21.59, SD= 3.77), 95% CI [.20, 3.52], $p= .019$. There was no statistically significant difference in total confidence score across roles, $F(2,145) = 2.27$, $p = .107$). A t-test was performed to compare perceived confidence across individuals who received training and those who did not. Respondents who received any training (M= 23.7, SD= 2.80) had significantly higher confidence scores than those who did not (M=20.7, SD= 2.94), 95% CI [1.93, 3.70], $t(146)=5.732$ $p<0.01$.

Respondents described barriers that could make it more difficult to provide psychological support. These included: (i) the need to prioritize the management of physical symptoms; (ii) limited time; (iii) communication challenges (iv) environment constraints and (v) family dynamics. Respondents reported that patients often have significant physical symptoms associated with their illness, which need to be prioritized over any coinciding psychological concerns.

> *"When a patient has physical pain but there is a psychological component- sometimes it feels as though we cannot get further with the psych distress until pain is better controlled, even though addressing the former will help the latter…"* (Respondent 39, Consultant)

There was a perception that effective psychological support requires more time than is available.

> *"Time is short... to be able to utilize nonpharmacological interventions effectively*" (Respondent 96, Nurse).

Communication challenges due to cognitive difficulties, language barriers or reluctance to discuss their situation made it more difficult to provide psychological support.

> *"Communication ability of patients i.e. if they have cognitive deficits or difficulty articulating makes it difficult to manage psychological distress."* (Respondent 32, Counsellor)

> *"Reluctance of patient to discuss their fears, accept their diagnosis/prognosis. Level of understanding of patients, language barriers."* (Respondent 63, Nurse)

A lack of privacy in inpatient settings can also hinder opportunities for the management of psychological concerns.

"It's really challenging to have sensitive and tender conversation in shared bays where cloth curtains provide visual privacy but by no means any audible privacy" (Respondent 87, Consultant)

Disagreements between patients and families about goals of care or how care is managed could also hinder access to psychological support.

"Difficulties arise…when the patient and family are not aligned in their thinking of the patients treatment. In some cases (families don't) consider it appropriate to consider counselling as an option" (Respondent 138, Doctor*)*.

In terms of facilitators of psychological support, many respondents reported that a therapeutic relationship with the patient and caregivers is the foundation of psychological care, and it is built based on trust, honesty and rapport.

"Building a trusting rapport allows [patients and families] to express their feelings, concerns and anxieties" (Respondent 17, Bereavement counsellor*)*

Referral of patients and caregivers for psychological support

More than half of respondents (54%, n=81/150) reported that guidelines for psychological support referrals were unclear within their organisations. One-third of respondents disagreed with the statement that they could arrange psychological support for patients when they needed to (n=55/150, 36%). Internally, respondents could most commonly refer patients to bereavement counsellors, complementary therapists, counsellors and the social work team for psychological support (Table 2). Under one-quarter of respondents (24%, n=36/151) could refer to an in-house clinical psychologist. Referring externally for psychological support was less common, with less than half of respondents referring to any external services for

psychological support. Where respondents did refer externally, referral to social work (48%, n=72/151) and clinical psychologists (43%, n=65/151) were most common (Table 3).

Qualitative data revealed referral challenges due to limited access to mental health specialists and lack of clarity in relation to referral processes. Limited access to mental health specialists could be due to part-time specialists:

> "*Only one part-time psychologist in the palliative care team*" (Respondent 52, Consultant)

Some respondents noted that patients were more likely to be referred for psychological support if they were cared for in a hospital setting; or if they had a cancer diagnosis.

This needs to be defined for readers that do not know EOLC = end of life care

> *"Having access to a regional psychologist for pall[iative] and EOLC that could be used by all Pall[iative] and EOLC services - there are often access if the patient is in an acute hosp[ital] setting but not if the patient is in a hospice."* (Respondent 21, Occupational therapist)

> *"..and very patchy support for those who don't have a cancer diagnosis"* (Respondent 52, Consultant)

Referral challenges were more often noted in relation to external referrals to specialist services:

> *"Referral procedures within own workplace are effective, but referral to external agencies can be difficult, especially mental health teams."* (Respondent 10, Nurse)

Respondents also reported lack of clarity around referral procedures and indicated a desire for further clarity on referral guidelines. There was also a perception that the waiting list for support could impact the likelihood of a referral being made:

*“We do not have clear referral processes or criteria and waits are perceived to be long, and I think this affects how often we might refer.” (*Respondent 39, Consultant)

*" Clear guidelines on when and whom to refer to, Standard referral documentation [would improve confidence] (*Respondent 114, Nurse)

Table 2. Internal and external referral for specialist psychological support (N=151)

**Internal referrals for psychological support**<br>(i.e. ability to refer to professionals within the hospice for psychological support)	**Number of respondents**	**%**
Bereavement Counsellor	94	62%
Complementary Therapist	92	61%
Counsellor	89	59%
Social Work	79	52%
Day therapies	50	33%
Doctor/nurse trained in psychological support	44	29%
Clinical Psychologist	36	24%
Allied Health Professionals (AHP)	36	24%
Chaplain	11	7%
Senior nurse manager	4	3%
Psychiatrist	2	1%
Other (Occupational therapist/Physiotherapist, family support worker, Senior nurse managers)	6	4%
**External referrals for psychological support**<br>(i.e. ability to refer to professionals external to the hospice for psychological support)		
Social Work	72	48%
Clinical Psychologist	65	43%
Psychiatrist	44	29%
Doctor/nurse trained in psychological support	31	21%
Allied Health Professionals (AHP)	29	19%
Counsellor	27	18%
Bereavement counsellor	26	17%
Complementary therapist	14	9%
Psychological support provided by day therapies	9	6%
General Practitioner	7	5%
Other	4	3%
Chaplain/ Pastoral care	2	1%

Not all respondents answered this question.

Education and training in providing psychological support

Overall, 70% (n=106/151) respondents reported having received training, either formal or informal, in providing psychological support to patients and families. Of these, the majority had received informal on-the-job training (82%, n=87/106) (Table 3). Among those who received training in formal psychological approaches (n= 55/151, 36%), training in mindfulness approaches (n=34), counselling (n=20) and Cognitive Behavioural Therapy (n=20) were most often reported.

add (CBT) to define for easier understanding later in qualitative analysis section

Most staff reported that training would enhance their provision of psychological support (95%, n=141/148). Less than half of the respondents reported their training was sufficient in psychological needs assessment (44%, n=67/151), management of patient psychological needs (45%, n=68/151), and management of caregivers' psychological needs (44%, n=67/151).

Table 3. Type of training received (N=151)

Training Received	n	% respondents who received any training (N=106)	% of all respondents (N=151)
Informal on the job training	87	82%	58%
In-house training as part of a general training day on a range of topics	60	55%	40%
Training in any formal psychological therapy	55	52%	36%
In-house training focused on psychological support	47	43%	31%
External training day or workshop focused on psychological support	44	40%	29%
A short, unaccredited course over a week or similar	17	16%	11%
Comprehensive, longer-term training, such as a certificate or diploma in psychological support/ interventions	14	13%	9%
Other	11	10%	7%

Qualitative findings indicated a desire for further training and supervision in providing psychological care, in particular in relation to supporting specific populations such as children and families.

"*Supervision following training in use of techniques to develop skills in delivering therapies such as CBT"* [would help]. (Respondent 112, Consultant).

"*Face to face training on giving psychological support to children and families within the remit of our role." [to improve participant confidence in the provision of psychological support]*. (Respondent 145, Nurse)

This needs to be defined for readers who do not know what CBT means or define when Cognitive Behavioral Therapy noted in previous section

Discussion

This study describes hospice staff perspectives, and their self-reported confidence and competence in providing key aspects of psychological support to patients and caregivers. Our findings indicate that hospice staff routinely assess for psychological concerns using their clinical judgement. Formal screening tools are not commonly used. Nearly all hospice staff agree that they are competent and confident in providing key elements of psychological support within the boundaries of their roles. Most staff strongly agree that they are confident in initiating and holding conversations around distressing topics. Lower levels of confidence and competence were found in relation to using formal screening tools and arranging and delivering appropriate psychological support. Time constraints were perceived as a barrier to psychological care, especially when physical symptoms need to be addressed. Despite good levels of self-reported confidence and competence in relation to discussing distressing issues, there was a consensus that training was insufficient, and further training would enhance practice.

Our findings reveal that most hospice staff routinely screen for psychological concerns based on clinical judgement. These findings support and extend findings from a national survey of palliative medicine physicians in the UK, which reported that 99% used the clinical interview to diagnose anxiety but most did not use formal tools.[25] Our findings also align with evidence from inpatient palliative care settings in the United States which found that common ways to screen for psychological distress were talking to family members (92%) and observing the patients' mood (90%); with only one-third using a formal screening tool for psychological distress.[26] Our finding contrasts with a 2020 UK survey of palliative care staff which reported that at least one-quarter of hospice staff did not screen or assess patients

for psychological problems.[14] The lower percentage in that study may be due to the way their question was framed (i.e. in context of referring to referral criteria) and the fact that responses were from staff in a range of roles, some of whom may not be involved in psychological needs assessment as part for their role. Our findings indicate that most clinical staff screen for psychological concerns, though this is not usually part of the patient's first assessment, in part due to time constraints and prioritisation of physical symptom management.

We found that most hospice staff report high levels of confidence and competence in relation to initiating and holding conversations on distressing issues. This suggests that many hospice-employed staff have enhanced communication skills and reflects the results from a survey of 181 hospice nurses based in the United States which concluded that hospice nurses perceive themselves as effective communicators with effective or very effective communication skills (85.6%).[27] That study also indicated that 'denial' is a challenge to communication, a finding we also identified in our qualitative data, which was another barrier to psychological assessment. While hospice staff feel confident in relation to discussing difficult topics with patients and families, further communication skills training focusing on how to approach denial in a palliative care context may be valuable.

Hospice-employed staff who received any psychological training felt more competent and confident in providing psychological support than those without training. These findings align with prior research linking psychological training with objective competence[28, 29] and perceived competence[30] and allude to the value of psychological support skills training in palliative care contexts. Key targets for training were psychological needs assessment and the delivery of psychological support. There is evidence that nurse-led psychological

interventions can be effective in enhancing the spiritual and psychological wellbeing of people receiving palliative care.[16, 28, 31-33] Life review, metacognitive therapy, Acceptance and Commitment Therapy, dignity therapy and Cognitive Behavioural Therapy have formed the basis for nurse-led interventions that show preliminary evidence of effectiveness in helping people manage psychological concerns and improve mental wellbeing. [28, 31-33] Training can be brief, but as noted in our findings and the wider literature, having access to supervision after initial training is essential so that skills are developed and sustained.[28, 30]

Our findings indicated a lack of clear patient referral guidelines for psychological support within hospice organizations. Similar findings were previously reported,[12] the only difference being that spiritual advisors featured more strongly in the earlier study with 78% of hospices reporting referring to a spiritual advisor for psychological support compared with only 8% reporting this in our study. The reason for the higher reported levels of access to a spiritual care provider in the earlier study is unclear, though may partly be due to use of the wider term "spiritual advisor" than "Chaplain" which may have more faith-based connotations.

Less than one-quarter (24%) of respondents could refer to an in-house clinical psychologist, while 43% could refer externally to a clinical psychologist. Similar findings were reported in a pre-pandemic UK national survey of hospice psychological support services.[12] In the latter, 19% of respondents had access to an in-house psychologist and 41% could refer externally. While these data do not take into account the actual whole time equivalent working hours of clinical psychologists in hospice settings, they reveal that in the UK, direct access to a clinical psychologist is available in a minority of hospices. Limited integration of

clinical psychologists in palliative care settings is evidenced in many countries including Canada, North America and Europe.[11, 34]

Implications for practice

Hospice-employed staff based in the UK report that they are competent and confident in providing basic levels of psychological support in line with Level 1 proposed by the NICE 2004 guidance.[8] This support is adequate for individuals who are not significantly impacted by anxiety, depression and adjustment problems resulting from a terminal diagnosis. However, access to more tailored psychological support for patients and families with more significant problems is limited due to little formal training in psychological support, and the lack of specialist mental health resource. One way to increase access to evidence based psychological support for patients and caregivers is to develop and implement psychological support skills training programmes for staff, with view to increasing in-house capacity, and ensuring that the clinical psychologist resource is protected for those who would most benefit. A lack of clarity around referral guidelines was identified as a problem here and elsewhere, thus a focus on clarifying local and national guidance is warranted. The NICE guidance is now over two decades old and needs to be revisited with view to focussing on the needs of the person rather than what the service can provide. Updated guidance that takes account of digital interventions, both self-directed and facilitated, is also needed to ensure that a wide range of support is available in a broad range of formats to meet the needs of a geographically distributed ageing population.

Future research questions

Future research is needed to explore the perspectives of patients and caregivers in relation to the psychological support provided to them by hospice staff and its effectiveness in addressing their needs. Research is also needed to investigate the value of using a formal assessment tool over clinical judgement alone. Research on the effectiveness and implementation of training programmes for hospice staff is also recommended. Brief training programmes, that can be delivered in a flexible format and include an ongoing supervision component may be of particular value. Lastly, hospice-employed staff are used to caring for people approaching end of life on a daily basis and are confident and competent in initiating difficult conversations with patients and families; health care professionals working in hospitals often encounter people approaching end of life,[35] but are unlikely to have the same experience and confidence in providing psychological support to people with an advanced illness. Exploring the perspectives and training needs of hospital-based healthcare professionals would be useful.

Advanced illness does not equal terminal illness and should be changed

Strengths & Limitations

A mixed-methods design enabled a comprehensive understanding of staff perceptions, combining qualitative insights with quantitative evidence. The involvement of stakeholders in the refinement of the online questionnaire enhanced content validity by confirming the relevance and suitability of questions to the needs and concerns of palliative care staff in providing psychological support, and ensuring no critical information was overlooked. The questionnaire was anonymous, minimising bias due to socially desirable responding. Unlike previous national surveys, the data was collected in May and June 2023, potentially representing a more current picture of how psychological support is provided compared with

studies which involved data collection before [12]or during the onset of the COVID pandemic. [14]

However, limitations should also be highlighted. The response rate was not possible to determine as we could not know how many eligible respondents had sight of the study invitation. Despite this the overall sample size was higher than that reported in recent studies examining the psychological care in hospice settings; with other studies reporting 116[12] and 140[14] respondents. The study relied on convenience sampling. It is possible that palliative care staff who are more inclined to provide psychological care, and perhaps more competent in it, may have been more likely to respond to the questionnaire than those less involved in psychological care. The competence and confidence scales were created for the purpose of the present study; while the questionnaire had face validity, it had not been psychometrically tested for validity and reliability.

Conclusion

Most hospice staff perceive themselves as competent and confident in providing basic psychological support. However, further training in the assessment and the delivery of psychological support is desired and would further enhance practice, especially in relation to caring for people with greater psychological support needs and navigating complex issues such as illness denial. Clearer guidance on referral for specialist psychological support is warranted.

Acknowledgements

The stakeholders played a key role in reviewing the questionnaire and making valuable suggestions. This enabled the research group to formulate a questionnaire that is relevant to the respondent group and representative of the concerns faced by staff in palliative care.

References

1. Ann-Yi S, Bruera E. Psychological Aspects of Care in Cancer Patients in the Last Weeks/Days of Life. Cancer Res Treat. 2022;54(3):651-60. doi: 10.4143/crt.2022.116.

2. Mitchell AJ, Chan M, Bhatti H, Halton M, Grassi L, Johansen C, et al. Prevalence of depression, anxiety, and adjustment disorder in oncological, haematological, and palliative-care settings: a meta-analysis of 94 interview-based studies. The Lancet Oncology. 2011;12(2):160-74. doi: http://dx.doi.org/10.1016/S1470-2045(11)70002-X.

3. Kolva E, Rosenfeld B, Pessin H, Breitbart W, Brescia R. Anxiety in Terminally Ill Cancer Patients. Journal of Pain and Symptom Management. 2011;42(5):691-701. doi: 10.1016/j.jpainsymman.2011.01.013.

4. Finucane AM, Swenson C, MacArtney JI, Perry R, Lamberton H, Hetherington L, et al. What makes palliative care needs "complex"? A multisite sequential explanatory mixed methods study of patients referred for specialist palliative care. BMC Palliat Care. 2021;20(1):18. Epub 2021/01/17. doi: 10.1186/s12904-020-00700-3. PubMed PMID: 33451311; PubMed Central PMCID: PMCPMC7809819 Marie Curie, a specialist palliative care provider with nine hospices across the UK, during the study. No other interests are declared.

5. Alexander SC, Ladwig S, Norton SA, Gramling D, Davis JK, Metzger M, et al. Emotional distress and compassionate responses in palliative care decision-making consultations. Journal of palliative medicine. 2014;17(5):579-84. Epub 2014/03/05. doi: 10.1089/jpm.2013.0551. PubMed PMID: 24588656; PubMed Central PMCID: PMCPMC4012632.

6. Antunes B, Rodrigues PP, Higginson IJ, Ferreira PL. Determining the prevalence of palliative needs and exploring screening accuracy of depression and anxiety items of the integrated palliative care outcome scale – a multi-centre study. BMC Palliative Care. 2020;19(1):69. doi: 10.1186/s12904-020-00571-8.

7. Gao W, Bennett MI, Stark D, Murray S, Higginson IJ. Psychological distress in cancer from survivorship to end of life care: Prevalence, associated factors and clinical implications. European Journal of Cancer. 2010;46(11):2036-44. doi: http://dx.doi.org/10.1016/j.ejca.2010.03.033.

8. National Institute for Clinical Excellence. Improving Supportive and Palliative Care for Adults with Cancer National Institute for Clinical Excellenc, 2004 March 2004. Report No.

9. National Comprehensive Cancer Network. NCCN Clinical Practice Guidelines in Oncology: Distress Management. Version 2.2023-December 22.2022. 2023.

10. National Coalition for Hospice and Palliative Care. Clinical Practice Guidelines for Quality Palliative Care. Richmond, Virginia: 2018.

11. Foo BMY, Sharpe L, Clayton JM, Wiese M, Menzies RE. The role of psychologists in supporting illness-related dying and death: A systematic mixed studies review. Clinical Psychology Review. 2024;110:102393. doi: https://doi.org/10.1016/j.cpr.2024.102393.

12. McInnerney D, Candy B, Stone P, Atkin N, Johnson J, Hiskey S, et al. Access to and adequacy of psychological services for adult patients in UK hospices: a national, cross-sectional survey. BMC Palliative Care. 2021;20(1):31. doi: 10.1186/s12904-021-00724-3.

13. Price A, Hotopf M, Higginson IJ, Monroe B, Henderson M. Psychological services in hospices in the UK and Republic of Ireland. J R Soc Med. 2006;99(12):637-9. Epub 2006/12/02. doi: 10.1177/014107680609901213. PubMed PMID: 17139068; PubMed Central PMCID: PMCPMC1676317.

14. Paley CA, Keshwala V, Farfan Arango M, Hodgson E, Chapman EJ, Birtwistle J. Evaluating provision of psychological assessment and support in palliative care: A national survey of hospices in England. Progress in Palliative Care. 2024;32(1):11-21. doi: 10.1080/09699260.2023.2286418.

15. Brook E, Chur-Hansen A, Chan L, Crawford G. Psychological service provision in Australian Palliative Care Services. Palliative and Supportive Care. 2023:1-7. Epub 2023/07/10. doi: 10.1017/S1478951523000925.

16. Zweers D, de Graaf E, Teunissen SC. Non-pharmacological nurse-led interventions to manage anxiety in patients with advanced cancer: A systematic literature review. International journal of nursing studies. 2016;56:102-13. Epub 2015/11/27. doi: 10.1016/j.ijnurstu.2015.10.022. PubMed PMID: 26607059.

17. Zweers D, de Graeff A, Duijn J, de Graaf E, Witteveen PO, Teunissen SCCM. Patients' Needs Regarding Anxiety Management in Palliative Cancer Care: A Qualitative Study in a Hospice Setting. American Journal of Hospice and Palliative Medicine®. 2019;36(11):947-54. doi: 10.1177/1049909119846844.

18. Health and Safety Executive. What is competence? 2024 [03 Septmeber 2024].

19. Gottlieb M, Chan TM, Zaver F, Ellaway R. Confidence-competence alignment and the role of self-confidence in medical education: A conceptual review. Medical education. 2022;56(1):37-47. Epub 2021/06/28. doi: 10.1111/medu.14592. PubMed PMID: 34176144.

20. Creswell JW, Plano Clark VL. Designing and Conducting Mixed Methods Research: Sage Publications.; 2018.

21. Younas A, Durante A. Decision tree for identifying pertinent integration procedures and joint displays in mixed methods research. J Adv Nurs. 2023;79(7):2754-69. Epub 2022/12/17. doi: 10.1111/jan.15536. PubMed PMID: 36524303.

22. Eysenbach G. Improving the quality of Web surveys: the Checklist for Reporting Results of Internet E-Surveys (CHERRIES). J Med Internet Res. 2004;6(3):e34. Epub 2004/10/09. doi: 10.2196/jmir.6.3.e34. PubMed PMID: 15471760; PubMed Central PMCID: PMCPMC1550605.

23. National Palliative and End of Life Care Partnership. Ambitions for Palliative and End of Life Care: a national framework for local action 2021-2026. 2021.

24. Gale NK, Heath G, Cameron E, Rashid S, Redwood S. Using the framework method for the analysis of qualitative data in multi-disciplinary health research. BMC Medical Research Methodology. 2013;13(1):117. doi: 10.1186/1471-2288-13-117.

25. Atkin N, Vickerstaff V, Candy B. 'Worried to death': the assessment and management of anxiety in patients with advanced life-limiting disease, a national survey of palliative medicine physicians. BMC Palliative Care. 2017;16(1):69. doi: 10.1186/s12904-017-0245-5.

26. Kozlov E, Eghan C, Moran S, Herr K, Reid MC. Palliative Care Providers' Practices Surrounding Psychological Distress Screening and Treatment: A National Survey. American Journal of Hospice and Palliative Medicine®. 2017;35(7):938-44. doi: 10.1177/1049909117743960.

27. Clayton MF, Iacob E, Reblin M, Ellington L. Hospice nurse identification of comfortable and difficult discussion topics: Associations among self-perceived communication effectiveness, nursing stress, life events, and burnout. Patient Education and Counseling. 2019;102(10):1793-801. doi: https://doi.org/10.1016/j.pec.2019.06.013.

28. Mannix KA, Blackburn IM, Garland A, Gracie J, Moorey S, Reid B, et al. Effectiveness of brief training in cognitive behaviour therapy techniques for palliative care practitioners. Palliat Med. 2006;20(6):579-84. Epub 2006/10/25. doi: 10.1177/0269216306071058. PubMed PMID: 17060250.

29. Moorey S, Cort E, Kapari M, Monroe B, Hansford P, Mannix K, et al. A cluster randomized controlled trial of cognitive behaviour therapy for common mental disorders in patients with advanced cancer. Psychological Medicine. 2009;39(5):713-23. Epub 2008/09/02. doi: 10.1017/S0033291708004169.

30. Cort E, Moorey S, Hotopf M, Kapari M, Monroe B, Hansford P. Palliative care nurses' experiences of training in cognitive behaviour therapy and taking part in a randomized controlled trial. Int J Palliat Nurs. 2009;15(6):290-8. Epub 2009/07/02. doi: 10.12968/ijpn.2009.15.6.42988. PubMed PMID: 19568216.

31. Kwan CWM, Chan CWH, Choi KC. The effectiveness of a nurse-led short term life review intervention in enhancing the spiritual and psychological well-being of people receiving palliative care: A mixed method study. International journal of nursing studies. 2019;91:134-43. Epub 2019/01/29. doi: 10.1016/j.ijnurstu.2018.12.007. PubMed PMID: 30690289.

32. Reb AM, Borneman T, Economou D, Cangin MA, Cope DG, Ma H, et al. A nurse-led intervention for fear of cancer progression in advanced cancer: A pilot feasibility study. European journal of oncology nursing : the official journal of European Oncology Nursing Society.

2020;49:101855. Epub 2020/10/30. doi: 10.1016/j.ejon.2020.101855. PubMed PMID: 33120211; PubMed Central PMCID: PMCPMC8493814.

33. Söderman A, Werkander Harstäde C, Östlund U, Blomberg K. Community nurses' experiences of the Swedish Dignity Care Intervention for older persons with palliative care needs – A qualitative feasibility study in municipal home health care. International Journal of Older People Nursing. 2021;16(4):e12372. doi: https://doi.org/10.1111/opn.12372.

34. Feldstain A. Psychosocial intervention in palliative care: What do psychologists need to know. J Health Psychol. 2024;29(7):707-20. Epub 2024/01/29. doi: 10.1177/13591053231222848. PubMed PMID: 38282369; PubMed Central PMCID: PMCPMC11141107.

35. Clark D, Armstrong M, Allan A, Graham F, Carnon A, Isles C. Imminence of death among hospital inpatients: Prevalent cohort study. Palliative Medicine. 2014;28(6):474-9. doi: 10.1177/0269216314526443.

Supporting Information

S1 File. Supplementary File 1.

S2 File. Supplementary File 2.

Figure 1

Click here to access/download;Figure;Figure 1.tif

Recommended model of professional psychological assessment and support

Self help and informal support

Level	Group	Assessment	Intervention
1	All health and social care professionals	Recognition of psychological needs	Effective information giving, compassionate communication and general psychological support
2	Health and social care professionals with additional expertise	Screening for psychological distress	Psychological techniques such as problem solving
3	Trained and accredited professionals	Assessed for psychological distress and diagnosis of some psychopathology	Couselling and specific psychological interventions such as anxiety management and solution-focused therapy, delivered according to an explicit theoretical framework
4	Mental health specialists	Diagnosis of psychopathology	Specialist psychological and psychiatric interventions such as psychotherapy, including cognitive behavioural therapy (CBT)

Figure 2

Click here to access/download;Figure;Figure 2.tif

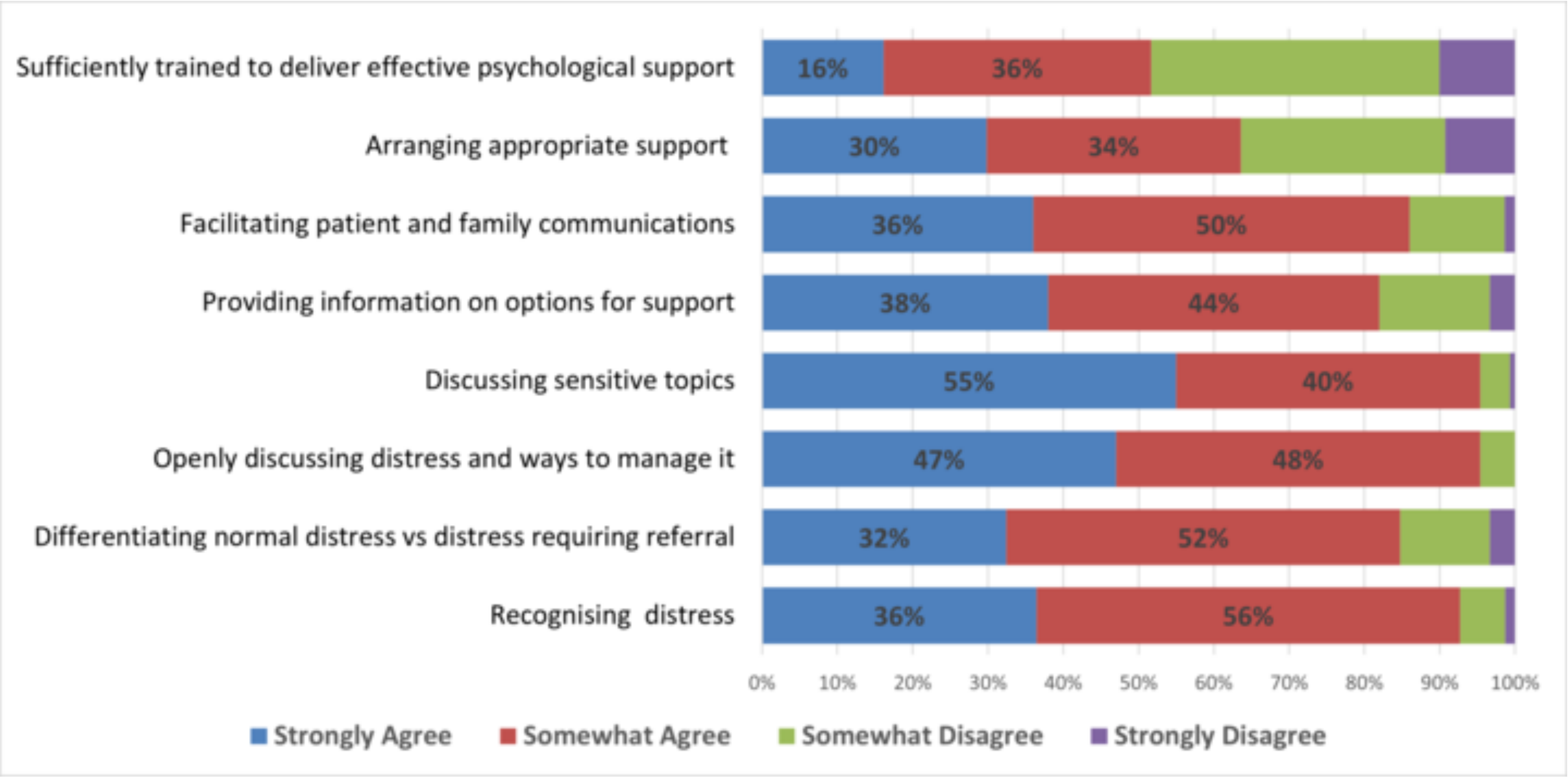

Figure 3

Click here to access/download;Figure;Figure 3.tif

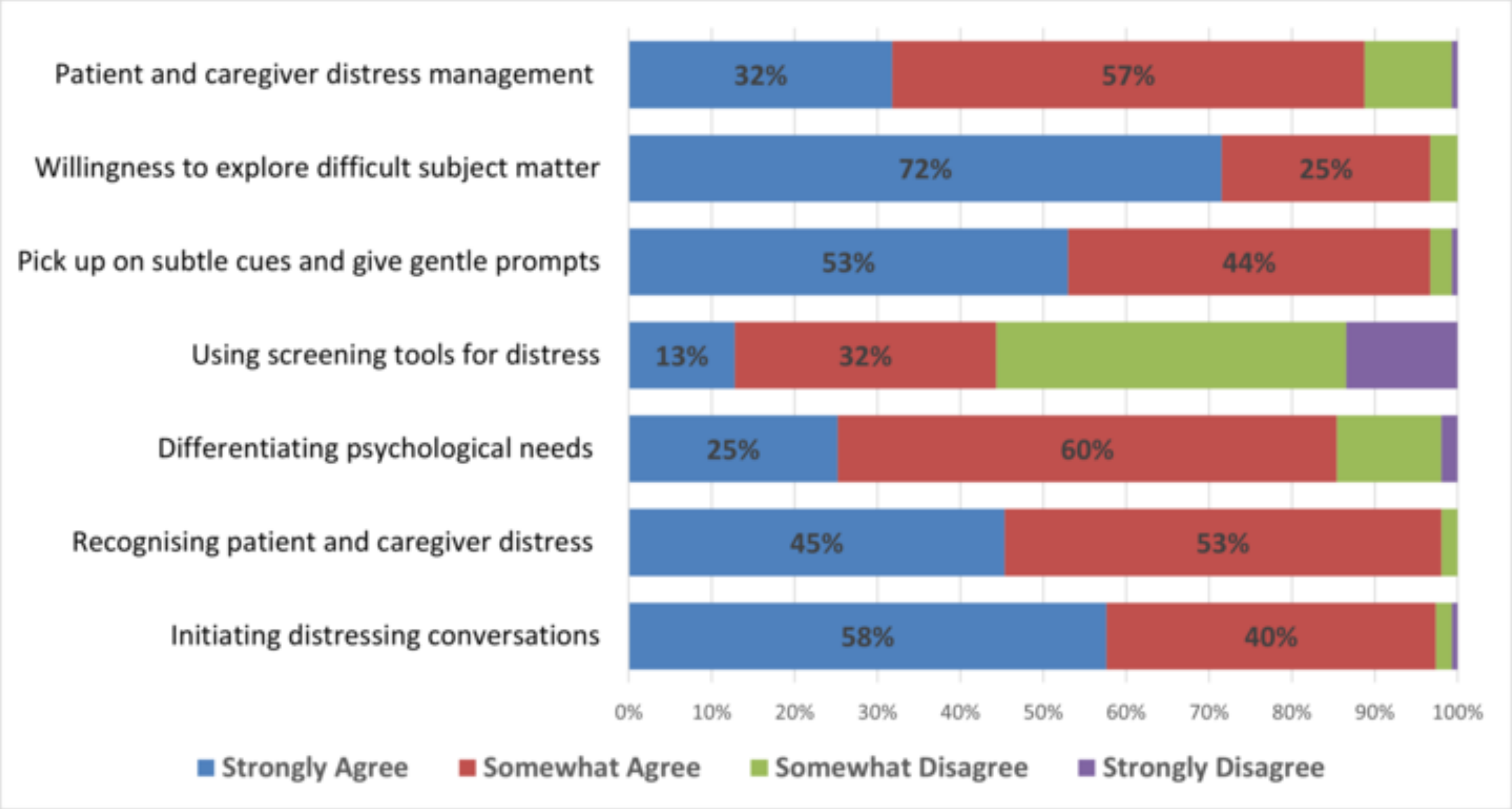

Click here to access/download

Supporting Information

S1_File.docx

Supporting Information File 2

Click here to access/download
Supporting Information
S2_File.docx
