# Supplementary material for: Providing psychological support to people impacted by terminal illness: A mixed methods study of hospice staff perceived competence, confidence and training needs
Source: PLoS One. 2025 Sep 24;20(9):e0331680. doi: 10.1371/journal.pone.0331680 (PMC12459835; doi:10.1371/journal.pone.0331680)
Supplement: Attachment — Submitted filename: Response to Reviewers.docx [file pone.0331680.s006.docx]

Dr Anne Finucane

University of Edinburgh Medical School
Doorway 6, Medical School
Teviot Place
EDINBURGH
EH8 9AG

United Kingdom

Editor

PLOS One

25^th^ May 2025

Dear Editor

# Thank you very much for considering our manuscript for publication.

# We are very grateful to the reviewers for their helpful comments and suggestions. We note our responses to each in turn below. We now also include an additional Supplementary File (S3) with our submission, which contains all anonymised study data.

We also confirm that further detail has been added on Ethics in line with journal requirement.

Thank you once again for your consideration.

Yours sincerely,

Anne Finucane

Fatema Zavery

Sulafa Fakhreddin

Sarah Huibregtse Van Loon

Mhairi McDougall

| **Reviewer 1 Comment** | **Response** |
| --- | --- |
| **General** | |
| Congratulations on your great work! You have studied a critical area in health.  There are some areas you should work on to improve the report. | Thank you!  We greatly appreciate these suggestions and respond to each in turn below. |
| Please rephrase the topic. Writing questionnaire in the topic seems quite inappropriate.    Include population intervention (if applicable), context and outcome in the topic. According to your study, it seems that you have studied perceived competence and confidence. | Thank you for this suggestion. We have now removed the term ‘questionnaire’. We already include the population (hospice staff) and now also include competence.  The title now reads: "*Providing psychological support to people impacted by terminal illness: A mixed methods study of hospice staff perceived competence, confidence and training needs*”. |
| Correct typo error and grammatical inconsistencies. | We have read through the paper and rectified the grammatical errors. |
| **Abstract** |  |
| Specify study design, rephrase eligible population, can use the term included participants, support results with figures | Thank you. The abstract has now been amended in line with the reviewer’s suggestion. |
| **Introduction** | |
| Cite who gave the projected data. | Apologies, we are unsure what is being asked for by the reviewer. |
| Link the information provided in the introduction, make it more organised. | The introduction has now been rewritten to make it more organised. |
| **Methods** | |
| was this a parallel mixed method? Please specify | This has now been specified in the design sub-section under the methods heading. |
| **Participants:**  who were your target population?    what was their total number in each of the hospices?    You mentioned that you excluded some members(like mental health professionals, and staff affiliated with NHS-based hospices) because you did not get the ethical approval for it. Did you wanted to collect data from these professionals but IRB/C did not allow you to?    What might be its adverse effect on the validity of the study?  Include the information about total number of participants in the study here rather than in the results | We have now clarified that hospice staff were our target population – see section on Participants - Eligibility.  Participant responses were anonymised. Therefore, the number of responses from each individual hospice is unknown. We now note this in the limitations (discussion).    We did not intend to include this group from the outset. The rationale for this has been added to the eligibility sub-section in the methodology. “*We did not seek to recruit from hospital-based palliative care units, as these are managed by the National Health Service and would have required additional research governance processes that could not be completed in the time available for the study”.*    Thank you – we now discuss this in Strengths and Limitations (Discussion).  This has now been moved to the Methodology section under Data collection. |
| You stated that you excluded the response with more than 50% missing data? how many questionnaire did you get with missing data less than 50%?, How did you manage it?    How did you manage your online data? | We now clarify this in the Methods section – under Data collection. We note that we only received one questionnaire with over 50% of questions unanswered and this was removed.  We describe how we managed our data in the Data Collection and analysis sections and have now added more detail. |
| What was the trend of your data? why did you choose t-test and ANOVA? | This information has now been added under the quantitative data analysis section. |
| Describe in detail about qualitative data analysis. How did you prevent bias in data analysis of qualitative studies? | This information has now been added under the qualitative data analysis section. |
| **Results** | |
| N= Population, n=sample; replace N with n in table heading and table | Noted. This has now been altered in all the tables in the manuscript. |
| No need to write number while describing tables, you can use only percentage but be clear where you keep it. | Noted. We have now deleted the numbers from the narrative text describing the data that can be seen in the tables. |
| What does "other" mean? was this term stated by participants?? | This was an open text box. We have now removed the inverted commas and added this detail: ‘‘*Other’ common concerns were noted by 26% of respondents in an open text box. These included fear of the future, spiritual distress, loss of control, worry about leaving family, and disrupted family dynamics.’* |
| Are the tables formatted and hyperlinked according to guidelines?  Tables need formatting | The tables have been reformatted. |

| **Reviewer 2 Comment** | **Response** |
| --- | --- |
| **General** |  |
| I would like to begin by highlighting the relevance and pertinence of the work presented. The study addresses a fundamental and often underestimated aspect of palliative care: the preparedness of hospice staff to offer psychological support to patients and families in contexts of terminal illness. | Thank you! |
| In the title it does not make sense to use the term questionnaire, since it does not indicate the research methodology. Therefore, I think it would be better to replace it by descriptive mixed methods study. | We have removed the term ‘questionnaire’. The title now reads: "*Providing psychological support to people impacted by terminal illness: A mixed methods study of hospice staff confidence and training needs”.* |
| Missing information on how to access the study data, please add the correct information when uploading the data to the platform instead of XXX. | Anonymised data is now provided in a new Supplementary File. See Supplementary File 3. |
| Explain abbreviations and acronyms throughout the text, such as NICE, NHS, JISC, EOLC.... | Thank you for the suggestion. All abbreviations have now been described in the manuscript. |
| **Introduction** |  |
| In the introduction, the sentence “In Australia, guidelines recommend a 0.15 full-time...” is not understood. Please explain what the 0.15 refers to? | I appreciate this statement is a bit confusing. We have now modified it to clarify the key point”..  *“In Australia, guidelines recommend that a psychologist is available, at least part-time, to provide support across community-based cancer services, acute settings and palliative care units* |
| Expand the introduction with the importance of training healthcare personnel in psychology skills and knowledge, especially in palliative care. | Thank you for pointing this out. We have now expanded on this towards the end of the Introduction. We note the value of training, while also pointing out that our study is important to help guide the development of such training. (Second last paragraph of the introduction). |
| **Methods** |  |
| The methodology lacks the calculation of the sample size, based on other studies we can know if we arrive at a sample calculation that is representative of the population under study. There are tools such as the GRANMO calculator that help to perform this type of calculation. | Thank you for highlighting this. We have now added an additional section, *sample size,* under the participants subtitle which details the rationale for the sample size. |
| It is not clear whether or not any software was used in the analysis of qualitative data, nor if data saturation was reached. | We analysed qualitative data in EXCEL. We did not use a specific software as the data was collected as part of a mixed method questionnaire, and we could manage the data easily in EXCEL. We now note this in the Qualitative data analysis section. |
| **Results** |  |
| In the results there are typos such as “ove to r” and in the respondent's response 21 occupational therapist. | We are unable to find the first typo stated.  The typo in respondent 21’s response has now been rectified. |
| The tables should be adjusted to the page size to improve the readability and comprehension of the data.  As well as the quality of the figures, since they look blurry and this hinders reading. | The tables have been adjusted as requested.  The figures have been revised and checked with the PACE tool, and are now of the required quality. |

| **Reviewer 3 Comment** | **Response** |
| --- | --- |
| **General** |  |
| The document lacks defined acronyms which need to be defined when first presented for readability and explanation to readers who have not seen or are aware of the acronym. | Thanks you for highlighting this, we now provide full terms alongside or instead of acronyms. |
| The document interchanges palliative care and hospice care and they are not the same thing.  More attention within the explanation needs to address that hospice clinicians provide palliative care to their patients and caregivers, but palliative care clinicians are not specifically hospice clinicians. Palliative care clinicians can take care of serious-illness patients that are not at the end of life or enrolled in hospice. | Noted. Thank you for pointing this out. We have now clarified this, and refer to *hospice* clinicians throughout where we are referring to our study sample and population. |
| There are some typographical errors and need to be corrected. | We have carefully read through the paper and rectified the typographical errors. |
