# Supplementary material for: Providing psychological support to people impacted by terminal illness: A mixed methods study of hospice staff perceived competence, confidence and training needs
Source: PLoS One. 2025 Sep 24;20(9):e0331680. doi: 10.1371/journal.pone.0331680 (PMC12459835; doi:10.1371/journal.pone.0331680)
Supplement: Attachment — Submitted filename: Response_to_reviewers_auresp_2.docx [file pone.0331680.s007.docx]

Dear Editor,

Thank you very much for considering our manuscript. We have considered each reviewer comment in turn and have amended our manuscript to increase clarity in response to these comments. We are very grateful to the reviewers for their thoughtful suggestions which have improved our manuscript. We also removed one reference which was no longer relevant, so the bibliography has been re-ordered very slightly.

Our point-by-point response to reviewers is outlined below.

Yours sincerely

Anne Finucane and Fatema Zavery

**Response to Reviewers**

| **Reviewer 1 Comment** | **Response** |
| --- | --- |
| **General** | |
| Overall: Congratulations! The manuscript looks better now. | Thank you! |
| **Abstract** |  |
| Setting /participants: free-text data was analysed thematically guided by which framework approach. Please specify | We now say ‘guided by the framework method’ in the abstract. This is a well-established approach which we reference in the Methods section (see paragraph headed Qualitative data analysis). |
| **Methods** | |
| Sample size: write about the sample size first and then only describe how you derived it. OR did you approached for complete enumeration? | Thank you. We have reordered this section so our target sample size appears first, followed by information on how it was derived. |
| Questionnaire development: why did you include demographic information at the last part of the questionnaire? | The rationale for adding demographic questions at the end has been included.  *“Placing demographic information at the end sought to ensure that participants focused first on the main questionnaire content, and were less likely to bias their responses in line with any perceived expectations linked with demographics or participant characteristics. Furthermore, by that point, participants would have already seen the full questionnaire and would likely feel assured there were no unexpected or uncomfortable questions, before providing demographic details.”*  The edit has been made in the Methods section, under the subheading, questionnaire development. |
| You mentioned that the tool has only face validity. Did you check for content validity of the tool? | We were unable to check for content validity given time and resource constraints to undertake this study. In our strengths and limitations section we clearly state that this measure has face validity only. In response to the reviewer's comment, we have now added a final sentence in the Strengths and Limitations section proposing that testing the validity of the competence and confidence scales would be a useful direction for future research. |
| Participant recruitment: how did you ensure that the non-elligible participants did not fill in the questionnaire? In the social media | We included a link to the participant information sheet in the online questionnaire, which highlighted eligibility, However, we cannot be 100% certain that non-eligible participants did not take part. We did not see any unusual patterns of data in our results. However, as this is a possibility, which we have now added in the limitations. |
| **Results** | |
| Table 1: what does N mean with regards to the table? | Thank you. This has now been changed to the lower-case n, which represents the sample size. |
| Figure 2: had statistically higher perceived competence scores than those who did not (M = 21.73, SD = 3.57), 95% CI [3.47, 6.18] (t (145) = 7.04, p <0.01)= is it necessay to include all the data in your result? | Noted. This has now been edited to only include the mean, t-test score and the p-value as this displays the difference in average values across two groups and the significance of these values. |
| Rewrite table according to the PLOS one guidelines. Have a look at other published articles. For example use small n instead for N as N means population, change format of tables | Noted. Table 1 titled sample characteristics on pages 13-15 and table 2 titled Internal and external referral for specialist psychological support on pages 21-23 have now been edited. |
| Table three: no need for another column, you can write n at each items of training received | Noted. The column has now been removed and the sample size has been detailed in the training received column.  The edit was made on page 24 under the subheading, education and training in providing psychological support. |
| **Discussion** | |
| well written | Thank you! |

| **Reviewer 2 Comment** | **Response** |
| --- | --- |
| **General** |  |
| I would like to extend my sincere congratulations on the work presented in your manuscript. The study reflects a commendable research effort, characterized by methodological rigor and a clear contribution to the body of knowledge in your field. | Thank you! |
